# Assessment of Melatonin-Cultured Collagen/Chitosan Scaffolds Cross-Linked by a Glyoxal Solution as Biomaterials for Wound Healing

**DOI:** 10.3390/antiox11030570

**Published:** 2022-03-17

**Authors:** Beata Kaczmarek-Szczepańska, Judith M. Pin, Lidia Zasada, Mauritz M. Sonne, Russel J. Reiter, Andrzej T. Slominski, Kerstin Steinbrink, Konrad Kleszczyński

**Affiliations:** 1 Department of Biomaterials and Cosmetics Chemistry, Faculty of Chemistry, Nicolaus Copernicus University, Gagarin 7, 87-100 Toruń, Poland; beata.kaczmarek@umk.pl (B.K.-S.); 296559@stud.umk.pl (L.Z.); 2 Department of Dermatology, University of Münster, Von-Esmarch-Str. 58, 48149 Münster, Germany; j_pin001@uni-muenster.de (J.M.P.); mauritzsonne@wwu.de (M.M.S.); kerstin.steinbrink@ukmuenster.de (K.S.); 3 UT Health Science Center, Department of Cellular and Structural Biology, San Antonio, TX 78229, USA; reiter@uthscsa.edu; 4 Department of Dermatology, Comprehensive Cancer Center, University of Alabama at Birmingham, Birmingham, AL 35294, USA; aslominski@uabmc.edu; 5 VA Medical Center, Pathology and Laboratory Medicine Service, Birmingham, AL 35294, USA

**Keywords:** biopolymers, scaffolds, glyoxal, melatonin, chitosan, cutaneous cells, wound healing

## Abstract

Chitosan (CTS) and collagen (Coll) are natural biomaterials that have been extensively used in tissue engineering or wound healing applications, either separately or as composite materials. Most methods to fabricate CTS/Coll matrices employ chemical crosslinking to obtain solid and stable scaffolds with the necessary porosity and mechanical properties to facilitate regeneration. In this study, we comparatively assessed the physicochemical properties of 3D scaffolds loaded with a cross-linker, glyoxal. Using a scanning electron microscope, we evaluated the microstructure of resultant matrices and their mechanistic testing by the determination of the compressive modulus (E_mod_), the maximum force (F_max_), thermogravimetric analysis (TG), Fourier Transform Infrared Spectroscopy–Attenuated Total Reflectance (FTIR-ATR), and proliferation rate in vitro using human epidermal keratinocytes and dermal fibroblasts cultured in presence of melatonin solution (10^−5^ M). We observed that enhanced content of collagen (50CTS/50Coll or 20CTS/80Coll compared to 80CTS/20Coll) significantly elevated the physicochemical capacities of resultant materials. Besides, presence of 5% glyoxal increased porosity, E_mod_ and F_max_, compared to scaffolds without glyoxal. Finally, keratinocytes and dermal fibroblasts cultured on subjected matrices in presence of melatonin revealed a prominently enhanced growth rate. This indicates that the combination of glyoxal and melatonin make it imperative to consider these materials as a promising approach for targeting skin tissue engineering or regenerative dermatology.

## 1. Introduction

Large molecules that consist of covalently bonded repeating monomer units called polymers can be obtained synthetically from fossil fuels such as coal, oil, natural gases, or they can be also isolated from natural sources. To date, there is an increasing imperative for new biopolymeric materials for cell-based transplantation, gene therapy, and tissue engineering; therefore, the enhancing environmental consciousness triggers us to introduce alternative renewable resources for the sustainable production of polymers [1]. Thus, compounds of natural origin are not harmful to the environment and this significantly increases their strengths [2]. Of note, biopolymers can be modified in order to change their functional capacities and this allows for the replacement of commonly used synthesized materials [3].

Polymer cross-linking consists of adding new substances to its basic structure and it has a great influence on its physicochemical properties [4]. Namely, this process is performed by the introduction of synthetic or natural substances containing reactive groups capable of forming covalent, intramolecular, or intermolecular bonds. It should be noted that cross-linking can be conducted either by immersing the polymer in the cross-linking agent or by adding the cross-linking agent directly to the solution [5,6,7].

Tissue engineering is a field of science that deals with the search for new methods of treating damaged tissues or entire organs. New methods of therapy for patients by creating new tissue scaffolds offer great opportunities to improve the state of regenerative medicine [8]. The selection of new biomaterial targeting the replacement patient’s tissue on the surface of the biomaterial is the key issue in terms of the entire treatment process.

Melatonin (*N*-acetyl-5-methoxytryptamine) is an indole-derivative, produced mainly by the pineal gland, and exerts ubiquitous occurrence in nearly every species [9]. Its pulsatile secretion with a peak during the night is considered a key mechanism by which the wake/sleep rhythm is orchestrated [10]. It is taken up by cells and may also be produced in mitochondria [11,12], where it improves mitochondrial metabolism by ATP enhancement or reduction of mitochondrial stress. Thus, melatonin activates anti-oxidative enzymes mediated via specific melatonin 1 (MT1) and MT2 membrane receptors [13,14,15,16,17,18,19,20].

Skin with hypodermis represents the largest body organ responding to environmental stresses [21,22,23]. Skin cells can produce and metabolize melatonin [24,25,26], and significant information has accumulated on the diverse function in this organ [27,28]. Thus, melatonin inhibits the tyrosinase activity, cell growth, and DNA synthesis of normal melanocytes [27,29], indicating a fundamentally different role than typical melanocyte peptide growth factors, which are induced upon UVB exposure, leading to increased skin pigmentation [30,31]. Furthermore, melatonin induces anti-oxidative and DNA repair mechanisms in skin cells after UVB exposure [32,33,34,35,36,37,38,39,40]. Significant high concentrations of melatonin have been detected in the bone marrow, cerebrospinal fluid, ovary, bile fluid, eye, lymphocytes, or skin [41]. Its amount is also differentially distributed in subcellular organelles due to its amphiphilic character [9,42,43]. Melatonin is able to efficiently protect intracellular organelles including proteins, enzymes, mitochondria, lipids, and the nucleus against oxidative damage [44,45]. Since mitochondria are target organelles for melatonin, it is reported to be responsible for mitochondrial or calcium homeostasis, preventing of neurodegenerative diseases or aging correlated to mitochondrial disturbances [46,47].

The objective of this study was to consider glyoxal as cross-linker for a chitosan/collagen mixture in the scaffold form accompanied by melatonin. The novelty of the study includes the use of glyoxal as a cross-linker for the biopolymeric mixture such as chitosan and collagen, in different weight ratios. Resultant scaffolds were assessed regarding physicochemical properties in order to consider their capacities for tissue regeneration.

## 2. Materials and Methods

### 2.1. Materials

Chitosan (CTS; DD = 78%, low molecular weight), glyoxal solution (40%), low glucose (1 g/L) MEM culture medium, MTT solution (5 mg/mL), PBS, acetic acid, ethanol, isopropanol, *L*-glutamine (200 mM), melatonin, and 0.05% trypsin/0.53 mM EDTA solution were purchased from Sigma (St. Louis, MO, USA). Fetal bovine serum was purchased from Thermo Fisher Scientific (Waltham, MA, USA). Collagen (Coll) used in this study was isolated from rat tail tendon by the procedures described previously [48].

### 2.2. Samples Preparation

CTS and Coll were dissolved in 0.1 M acetic acid (the final solution: 1%). Subsequently, CTS/Coll solutions were prepared as 80/20, 50/50, and 20/80 (*w/w*) ratio on the magnetic stirrer. Glyoxal solution was added (5% *w/w*) and the resultant scaffolds were placed into 24-wells plates, frozen, and lyophilized. Next, they were assessed for physicochemical properties as well as proliferation analysis.

### 2.3. Scanning Electron Microscope (SEM)

The internal morphology of the subjected scaffolds was studied using a scanning electron microscope (SEM; LEO Electron Microscopy Ltd., Cambridge, UK), where samples were gold-covered to form the conductive surface for the electron beam interaction and respective images were made accordingly for each examined scaffold with a resolution of 500 µm.

### 2.4. Stability Test

Dry scaffolds with known weight were immersed in PBS solution (pH = 7.4). After 1 and 24 h, subjected scaffolds were removed from the solution, frozen, and lyophilized. Obtained matrices were weighed and their weight change was determined as a result of the scaffolds immersion.

### 2.5. Mechanical Testing

Mechanical properties were measured using a mechanical testing machine (Shimadzu EZ-Test EZ-SX, Shimadzu, Kyoto, Japan). Analysis was carried out by a scaffolds compression with the initial force of 0.1 N and speed of 5 mm min^−1^. The Young modulus and the maximum force were determined with the Trapezium X Texture program.

### 2.6. Thermal Properties

Thermogravimetric assessment (TG) was conducted using a TA Instruments SDT 2960 Simultaneous TGA–DTA at a heating rate from 25 °C to 600 °C, in nitrogen (heating rate of 10 °C/min). Respective spectra were analyzed using of TA Universal Analysis software to determine the maximum temperatures and the enthalpy of the process.

### 2.7. Fourier Transform Infrared Spectroscopy—Attenuated Total Reflectance

FTIR-ATR analysis was made for each kind of sample. Dry scaffolds were scanned in the range 4000–600 cm^−1^ with 4 cm^−1^ resolution (64 scans) with the use of the Nicolet iS10 spectrophotometer equipped with an attenuated total reflectance (FTIR-ATR) device with a germanium crystal (Nicolet iS10, Thermo Fisher Scientific, Waltham, MA, USA) using OMNIC software.

### 2.8. Cell Culture

Human epidermal keratinocytes (NHEK) and dermal fibroblasts (NHDF) were supplied by PromoCell (Heidelberg, Germany) and American Type Culture Collection (ATCC) (Manassas, VA, USA), respectively. NHEK were maintained in Keratinocyte Growth Medium 2 supplemented with 1% Pen/Strep solution, while NHDF were cultured in MEM medium supplemented with 10% (*v/v*) heat-inactivated fetal bovine serum, 2 mM *L*-glutamine, and 1% (*v/v*) streptomycin-penicillin solution. Cells were seeded on 24-well plates at the density of 0.5 × 10^5^ cells/well to allow the cells to attach to the subjected 5% glyoxal-loaded scaffolds for 24 h. Comparatively, cells were cultured in the presence of melatonin, which was prepared in EtOH/1 × PBS to yield 10^−2^ M stock solution; then, they were subsequently dissolved in culture medium to the final working concentration of 10^−5^ M. Cells were cultured for 96 h and the culture medium was refreshed every 48 h. Cell viability was assessed using the MTT assay.

### 2.9. Cell Viability Assay

The MTT assay was performed as stated in the previously described procedure [49]. MTT solution (5 mg/mL in 1 × PBS) was added to each well, and cells were incubated for 3 h in a humidified atmosphere of 5% CO_2_ at 37 °C. The crystals of formazan were dissolved in isopropanol/HCl, and absorbance was measured at *λ* = 595 nm using the BioTek ELx808™ microplate reader (BioTek Instruments, Inc., Winooski, VT, USA).

### 2.10. Statistical Analysis

Data were expressed as the pooled means + standard deviations (S.D.) of six experiments (*n* = 6). Significant differences were determined using the ANOVA or the Student’s *t*-test (GraphPad Prism 7.05, La Jolla, CA, USA). We presented the obtained results as a percentage of the control sample and *p* < 0.05 was considered as statistically significant. 

## 3. Results

### 3.1. Scanning Electron Microscope (SEM)

**Figure 1:**
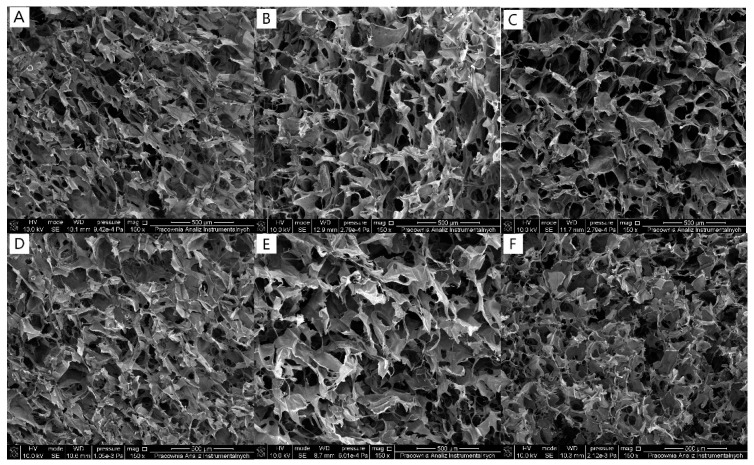
SEM images of 80CTS/20Coll (**A**), 80CTS/20Coll+5%G (**B**), 50CTS/50Coll (**C**), 50CTS/50Coll+5%G (**D**), 20CTS/80Coll (**E**), and 20CTS/80Coll+5%G (**F**) (magnification: 150×).

The morphology of scaffolds is an important factor to be considered for the biomedical application of designed materials. All scaffolds based on chitosan and collagen mixture with or without glyoxal supplementation revealed a porous structure with interconnected pores. Thus, the glyoxal addition enhanced the porosity of the scaffolds; however, it did not changed the shape of pores (Figure 1A–F).

### 3.2. Stability Test

Scaffolds based on natural polymers are able to swell in aqueous conditions [50]. Herein, all samples revealed their weight increase. It is related to the swelling process, which occurs after the scaffolds’ immersion in the PBS solution. The increase of collagen content results in elevated weight change, and this suggests that collagen has a higher ability to swell than chitosan itself. However, for each type of the investigated samples, additional loading with glyoxal resulted their weight decrease. Glyoxal, acting as a cross-linker, stabilizes the biopolymeric structure, and the swelling ratio of the scaffolds is significantly lower. Moreover, scaffolds with and without glyoxal showed that their weight changes were lower after 24 h compared to 1 h (Table 1), and this may suggest that the degradation process of the samples was initiated.

### 3.3. Mechanical Testing

**Figure 2:**
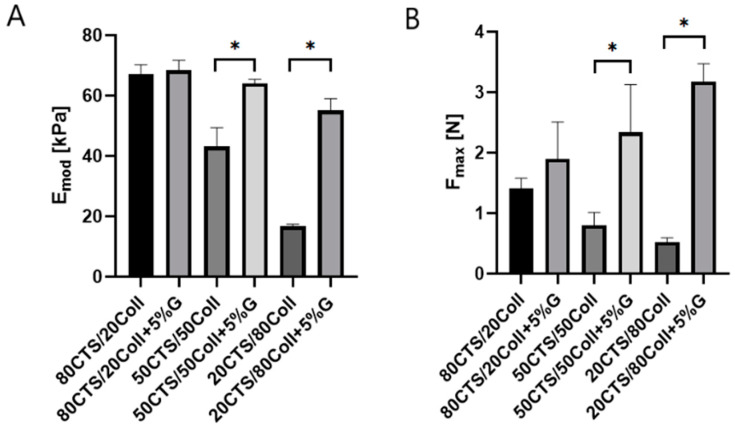
The compressive modulus (E_mod_) (**A**) and maximum force (F_max_) (**B**) obtained for the scaffold during the compression; * *p* < 0.001.

The cross-linking process may be carried out to improve the mechanical properties of the material, as it has been shown previously [51]. Thus, considering the chitosan/collagen mixtures, it was observed that enhanced chitosan content triggered the higher mechanical parameters (Figure 2). The glyoxal addition is considered as a cross-linker of the chitosan/collagen-based materials. Indeed, our study of subjected scaffolds revealed significant differences in terms of mechanical capacities between non-cross-linked samples (50CTS/50Coll and 20CTS/80Coll) containing 5% glyoxal and those where this cross-linker was present. Furthermore, cross-linked scaffolds showed a significantly higher compressive modulus (E_mod_) (Figure 2A) and maximum force (F_max_) (Figure 2B) compared to materials without glyoxal. Besides, the increasing content of collagen in subjected scaffolds from 80CTS/20Coll via 50CTS/50Coll until 20CTS/80Coll caused the prominent reduction of E_mod_ and F_max_.

### 3.4. Thermal Properties

During the thermal decomposition for each scaffold, three-stage process was observed (Table 2). The first and second ones are related to the release of water molecules structurally bonded to the material components [52]. Namely, the addition of glyoxal decreased the T_1_ for each CTS/Coll ratio; however, T_2_ decreased only for the 80CTS/20Coll composition. The third stage is related to the denaturation of polymers caused by high temperatures. The addition of glyoxal as a cross-linker did not affect the increase of T_3_. This suggests that the thermal stability of the material was not significantly improved. The highest maximum temperature of the third stage was noticed for the 80CTS/20Coll composition, without glyoxal, and with the 5% addition, respectively. Thus, this may suggest that glyoxal is more effective for materials with a higher amount of polysaccharides than protein.

### 3.5. Fourier Transform Infrared Spectroscopy–Attenuated Total Reflectance

**Figure 3:**
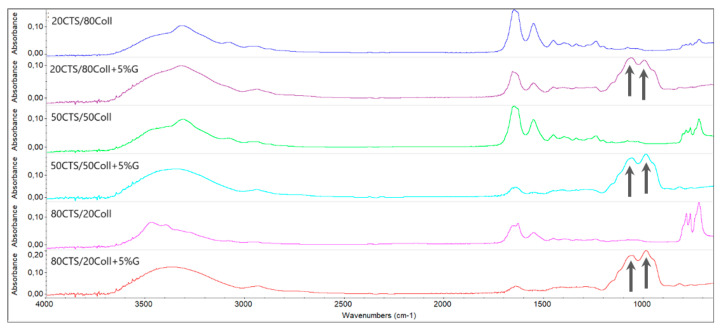
FTIR-ATR spectra obtained for all tested samples where the characteristic peaks are visible upon incorporation of 5% glyoxal (arrows).

Obtained FTIR-ATR spectra revealed changes triggered by the presence of glyoxal in the subjected scaffolds (Figure 3). The addition of this cross-linker induced the appearance of new peaks present in the spectra around 1061 and 988 cm^−1^, representing the skeletal vibrations of the C–O bonds. Thus, these peaks are characteristic for chemical compounds such as glyoxal, which are absent within the matrices without this cross-linker (Figure 3). Besides, there were visible changes in the shape of peaks around 3340 cm^−1^ between the cross-linked and non-cross-linked samples. This could be connected with the fact that the –OH and –NH groups that provide these peaks interact with the cross-linker; however, their position was not significantly shifted and it may be assumed that only hydrogen bonds are formed. Additionally, peaks in the range of 767–723 cm^−1^ from C–H out-of-plane deformation are not present after the addition of glyoxal since it stabilizes the polymeric structure; however, they may be observed on the spectra for scaffolds without glyoxal.

### 3.6. Cellular Assessments Using Human Epidermal Keratinocytes and Dermal Fibroblasts

**Figure 4:**
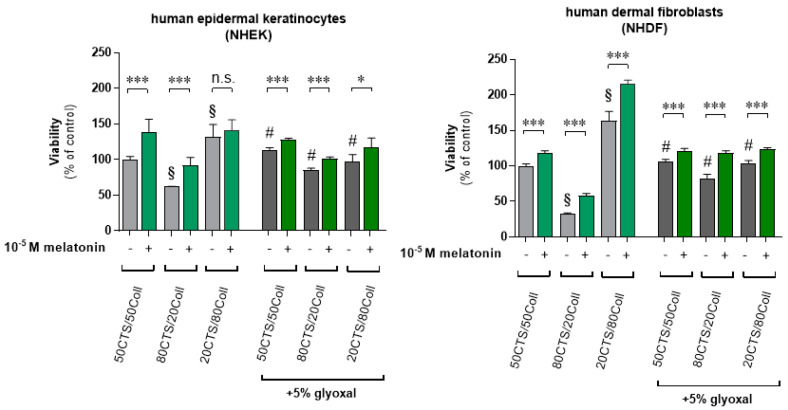
Human epidermal keratinocytes (NHEK) and human dermal fibroblasts (NHDF) were seeded on CTS/Coll scaffolds alone or containing 5% glyoxal in comparison to the culture medium including 10^−5^ M melatonin, cultured for 96 h, and viability was assessed using the MTT assay as described in Materials and Methods. Data are presented as mean + S.D. (*n* = 6), expressed as a percentage of the control sample (cells cultured on scaffolds without glyoxal and without melatonin). Statistically significant differences were indicated as follows; ^§^ *p* < 0.01: vs. 50CTS/50Coll sample, # *p* < 0.01: comparison of the corresponding samples containing 5% glyoxal and scaffolds alone, and corresponding statistical comparison as * *p* < 0.05, *** *p* < 0.001, n.s.-not significant.

Herein, we assessed the proliferation rate of human epidermal keratinocytes (NHEK) and dermal fibroblasts (NHDF) cultured on various CTS/Coll ratio scaffolds compared to the 5% glyoxal-loaded ones. The pattern of regulation was similar in both cell lines (Figure 4). Thus, 80CTS/20Coll led to a 39% (NHEK) and 67% (NHDF) drop, and 20CTS/80Coll caused a 32% (NHEK) and 64% (NHDF) enhancement of viability vs. the 50CTS/50Coll samples. Comparatively, cells cultured in the 10^−5^ M melatonin-supplemented culture medium significantly elevated cell viability for the respective scaffolds by 39% (50CTS/50Coll), 30% (80CTS/20Coll), and 10% (20CTS/80Coll) for NHEK, and by 18% (50CTS/50Coll), 25% (80CTS/20Coll), and 52% (20CTS/80Coll) for NHDF. Furthermore, scaffolds enriched by glyoxal (without melatonin) revealed a significant increase of cell viability compared to the respective CTS/Coll scaffolds without glyoxal itself. 

Namely, NHEK cultured on glyoxal-enriched scaffolds showed 14% and 24% enhancement for 50CTS/50Coll and 80CTS/20Coll, respectively, and a 36% drop for the 20CTS/80Coll sample. Similarly, proliferation of NHDF increased significantly by 10% (50CTS/50Coll) and 49% (80CTS/20Coll) against a 59% decrease of viability for 20CTS/80Coll. Finally, scaffolds containing 5% glyoxal and cultured cells in the presence of melatonin showed significant elevations of cell proliferation, indicating melatonin’s capacity towards skin re-epithelization.

## 4. Discussion

Scaffolds dedicated for biomedical application should have a porous structure to allow for the oxygen flow since oxygen distinctly accelerates wound healing processes. Thus, it is involved in cell proliferation, angiogenesis, and protein synthesis, which are all required for restoration of tissue integrity [53]. Scaffolds obtained in this study showed a porous structure with interconnected pores, and their application on the skin surface would not inhibit the flow of gases. It has been studied previously that glyoxal exerts the ability to react with hydroxyl and amine groups [54,55,56]. Both of them are present in chitosan as well as in collagen. Glyoxal has cross-linking ability via acetal formation between the aldehyde groups of glyoxal and the hydroxyl groups of the glucosamine units of chitosan, or via Schiff’s base formation between the free amino groups of chitosan or collagen and the aldehyde groups of glyoxal [56]. To consider the influence of a cross-linker on the materials’ properties, the zeta potential can be elucidated. The use of glyoxal results in the decrease of positive zeta potential values [57]. It can be explained that some of the amine groups are occupied by the cross-linker, which is glyoxal itself.

Scaffolds obtained from natural polymers possess the ability to swell but also degrade in aqueous conditions [58]. The balance between those two processes depends on the time of immersion and the type of solvent. Scaffolds based on chitosan and collagen revealed a high ability to swell in the initial stage of the experiment. However, the weight change decreased after 24 h, which indicates the degradation process. The addition of glyoxal resulted in the inhibition of those two processes insofar as the weight change was lower than for the scaffolds without cross-linker. Similar results were obtained by Perez-Puyana [59], who showed that the cross-linking decreased the swelling ratio of the scaffolds based on chitosan and collagen.

The low mechanical parameters of the biopolymeric scaffolds affect the ability to bind water molecules, which causes the increase of the materials’ flexibility [60]. Thereby, there is a need to search for compounds that can be added to newly introduced materials to improve mechanical properties, and cross-linkers may be proposed as such substances. However, it is important to consider their influence on polymers and detect the formation of bonds and/or interactions [61]. The most effective are those cross-linkers, which are able to form covalent bonds with functional groups of the polymeric chain. Nevertheless, strong hydrogen bonds formed between material components can also improve the mechanical properties of scaffolds. For instance, and comparatively, it was reported that the chitosan/collagen mixture can be cross-linked by genipin [62], EDC/NHS [63], or dialdehyde starch [64]. It was additionally described that glyoxal is an effective cross-linker for chitosan [65], poly(vinyl alcohol) [66], and starch [67]. Indeed, our studies showed that glyoxal could be proposed as an effective cross-linker for chitosan/collagen matrices. Namely, the addition of glyoxal to the mixture in the 80CTS/20Coll ratio did not provide a significant improvement of the mechanical properties. Contrarily, a statistically significant increase of the compression modulus and maximum force was observed for ratios 50CTS/50Coll and 20CTS/80Coll. This confirms that glyoxal is a more effective cross-linker for materials with a higher content of collagen. On one side, it resulted in the presence of hydroxyl groups in the collagen structure, which, on the other side, are able to form hydrogen bonds with glyoxal, and they are stronger than hydrogen bonds with amine groups. Furthermore, thermal stability was also noticed for the composition of the scaffolds based on 50CTS/50Coll and 20CTS/80Coll. These results are in agreement with previous report described by Wang et al. [68]. The authors assessed subjected hydrogels based on the various chitosan/collagen weight (*w/w*) composition as follows: 75/25, 50/50, 25/75, and 0/100. Results revealed that increased collagen content improved cell viability, and matrices were stiffer compared to those which did not contain the respective cross-linker. Similar responses were observed in terms of the FTIR assessment, where Beşkardeş et al. [69] or Grabska-Zielinska et al. [70] indicated appearance of characteristic peaks followed by the incorporation of the respective cross-linker, which is in accordance with our study when glyoxal was added to the subjected matrices.

Besides, we comparatively assessed the impact of subjected scaffolds loaded with glyoxal on the proliferation ratio of human cutaneous cells (human epidermal keratinocytes, NHEK, and dermal fibroblasts, NHDF) cultured with the addition of melatonin alongside our previous report [71]. The presence of 10^−5^ M melatonin prominently enhanced the growth ratio, indicating the proliferating capacity of melatonin itself, and this effect was also notable when glyoxal was present. A similar pattern of regulation in terms of proliferation ratio was noticed in our previous report [71]. We observed that cells cultured on scaffolds loaded with melatonin revealed an enhanced growth rate, and it was visible within human keratinocytes and human fibroblasts, but also in human melanoma cells used as reference models [62]. It should still be added that melatonin acts as a “double-edged sword” depending on the current cell conditions [72]. Thus, we know that melatonin protects cells in terms of maintaining mitochondrial homeostasis and scavenging ROS under stress conditions [32,33,34,35,36,37,38,39,40]. On the other hand, in normal conditions, i.e., without external stress factors, melatonin elevates proliferation rate and it significantly enhances oxidative phosphorylation. Based on our previous report [24], we know that melatonin applied exogenously may enhance the skin barrier, the expression of involucrin and keratins, and it increases the proliferative activity of keratinocytes in ex vivo skin explants, which is in agreement with previous earlier observations on murine skin [73]. Apart from this, melatonin can also accelerate skin wound healing [74,75,76,77] and can improve the antimicrobial action of wound dressing [75]. Despite melatonin’s anti-oxidative actions and the information described above, its physiological key role of a tightly controlled burst of reactive oxygen species production in tissue repair and regeneration [78,79], one of the functions of the melatoninergic system [28,80], may also be to control reactive oxygen species availability during wound healing. Thus, brand new studies [81,82,83,84,85] confirmed that melatonin may exert a beneficial role on epidermal barrier formation, indicating altogether that this indoleamine in combination with glyoxal could be considered as a safe solution, improving wound healing potential.

## 5. Conclusions

Chitosan and collagen could be lyophilized to obtain forms known as 3D scaffolds. On the other hand, glyoxal solution is reported as a good cross-linking agent for 3D materials. Thus, cross-linking with glyoxal solution modified the physicochemical properties of the biomaterials. The presence of glyoxal in the scaffolds results in the decrease of the swelling ratio and slightly reduces the thermal stability. Namely, it revealed elevated porosity and increased water content, accelerating tissue regeneration. Besides, the presence of glyoxal does not affect cell proliferation of cutaneous cells, i.e., human epidermal keratinocytes or human dermal fibroblasts, which were additionally assessed in the presence of melatonin. This indoleamine significantly enhanced cell proliferation when compared either to scaffolds without 5% glyoxal and to those containing this cross-linker. To sum up, 50CTS/50Coll and 20CTS/80Coll scaffolds were found to be the most effective and enrichment with glyoxal could be an effective alternative to the current methods of cross-linkage and a future tool in terms of designing biomaterials for therapeutic approaches for skin tissue engineering or wound healing.

## Figures and Tables

**Table 1:** The weight change of scaffold after 1 h and 24 h immersion in PBS.

Specimen	1 h [%]	24 h [%]
80CTS/20Coll	2730 ± 25	2110 ± 28
80CTS/20Coll+5%G	1750 ± 29	1560 ± 22
50CTS/50Coll	3215 ± 38	2877 ± 34
50CTS/50Coll+5%G	2890 ± 44	2540 ± 29
20CTS/80Coll	3980 ± 31	3570 ± 41
20CTS/80Coll+5%G	3470 ± 35	3109 ± 27

**Table 2:** Parameters of the thermal decomposition of the scaffolds for the maximum temperature of the obtained peaks on TG-DTG spectra.

Specimen	T_1_ [°C]	T_2_ [°C]	T_3_ [°C]
80CTS/20Coll	62.69	172.28	285.53
80CTS/20Coll+5%G	54.54	165.85	284.68
50CTS/50Coll	55.49	161.17	282.51
50CTS/50Coll+5%G	51.41	168.79	281.68
20CTS/80Coll	55.76	160.27	281.28
20CTS/80Coll+5%G	51.47	160.85	277.05

## Data Availability

Data is contained within the article.
